# Transcriptomic and anatomic profiling reveal the germination process of different wheat varieties in response to waterlogging stress

**DOI:** 10.1186/s12863-020-00901-y

**Published:** 2020-08-28

**Authors:** Changwei Shen, Jingping Yuan, Hong Qiao, Zijuan Wang, Yuanhai Liu, Xiujuan Ren, Fei Wang, Xing Liu, Ying Zhang, Xiling Chen, Xingqi Ou

**Affiliations:** 1 School of Resources and Environmental Sciences, Henan Institute of Science and Technology, Xinxiang, 453003 China; 2 School of Horticulture and Landscape Architecture, Henan Institute of Science and Technology, Xinxiang, 453003 China; 3 Xinxiang Nongle Seed Industry Co. Ltd, Xinxiang, 453003 China; 4 School of Life Science and Technology, Henan Institute of Science and Technology, Xinxiang, 453003 China

**Keywords:** Waterlogged, Wheat, Endosperm, Anatomical structure, Transcriptome, Differentially expressed gene

## Abstract

**Background:**

Waterlogging is one of the most serious abiotic stresses affecting wheat-growing regions in China. Considerable differences in waterlogging tolerance have been found among different wheat varieties, and the mechanisms governing the waterlogging tolerance of wheat seeds during germination have not been elucidated.

**Results:**

The results showed no significant difference between the germination rate of ‘Bainong 207’ (BN207) (after 72 h of waterlogging treatment) and that of the control seeds. However, the degree of emulsification and the degradation rate of endosperm cells under waterlogging stress were higher than those obtained with the control treatment, and the number of amyloplasts in the endosperm was significantly reduced by waterlogging. Transcriptomic data were obtained from seed samples (a total of 18 samples) of three wheat varieties, ‘Zhoumai 22’ (ZM22), BN207 and ‘Bainong 607’ (BN607), subjected to the waterlogging and control treatments. A comprehensive analysis identified a total of 2775 differentially expressed genes (DEGs). In addition, an analysis of the correlations among the expression difference levels of DEGs and the seed germination rates of the three wheat varieties under waterlogging stress revealed that the relative expression levels of 563 and 398 genes were positively and negatively correlated with the germination rate of the wheat seeds, respectively. Gene Ontology (GO) and Kyoto Encyclopedia of Genes and Genomes (KEGG) analyses showed that the difference in the waterlogging tolerance among the three wheat varieties was related to the abundance of key genes involved in the glycolysis pathway, the starch and sucrose metabolism pathway, and the lactose metabolism pathway. The alcohol dehydrogenase (ADH) gene in the endosperm of BN607 was induced immediately after short-term waterlogging, and the energy provided by the glycolysis pathway enabled the BN607 seeds to germinate as early as possible; in addition, the expression of the AP2/ERF transcription factor was upregulated to further enhance the waterlogging tolerance of this cultivar.

**Conclusions:**

Taken together, the results of this study help elucidate the mechanisms through which different wheat varieties respond to waterlogging stress during germination.

## Background

Due to high rainfall, irrigation practices and/or poor soil drainage, waterlogging annually affects large areas of farmlands worldwide, and these effects result in anoxic (absence of O_2_) soils and severe hypoxia or anoxia within crop roots. Hypoxia caused by waterlogging can inhibit the growth of crop roots and stems, the accumulation of dry matter and the final yield [1]. Oxygen is crucial for generation of the ATP needed to drive secondary energy-dependent ion transport. The reduced uptake of nutrients under hypoxic conditions is mainly due to inefficient oxygen transport down to the root [2]. Relevant studies have shown that waterlogging might affect the hormone content in wheat roots and stems, possibly by impacting the production of ethylene [3], and then reduce the absorption of mineral nutrients by plant roots [4]. Wheat is an important food crop widely planted throughout the world and is ranked first in terms of harvest area among the three major food crops (rice, wheat and corn). China is the largest wheat-producing and wheat-consuming country in the world, and wheat is of great significance to China’s food security and Chinese farmers’ incomes. According to the World Food and Agriculture Organization (FAO), approximately 10% of the world’s land area is affected by waterlogging to varying degrees [5]. In the Mediterranean region, the germination and growth stages of winter wheat are vulnerable to waterlogging because approximately 40% of the annual rainfall occurs during the sowing period of winter wheat [6]. In addition, the uneven terrain or poor drainage systems of farmlands easily cause waterlogging in the soil, which often leads to a lack of oxygen supply to the soil and the inhibition of seed germination, and these effects reduce the germination rate (GR) of wheat [7] and result in reductions in wheat production.

Both the timing and duration of waterlogging affect crop yields. In the case of wheat, the two stages at which waterlogging is most detrimental to wheat yields after germination and emergence are the seedling stage and the flowering stage [8]. Setter and Waters [8] found that waterlogging was most damaging during pre-emergence because it mostly results in the killing of seeds and/or very young seedlings. Water near the soil surface floods all the tissues of wheat seeds/seedlings, including the coleoptiles [9, 10]. In contrast to rice, wheat seeds cannot germinate under anoxic conditions. Specifically, wheat seeds under hypoxia cannot decompose starch into sugar and thus cannot germinate. The supply of exogenous sugar results in germination of 84% of seeds, but only slight root elongation (a few millimetres) and no coleoptile growth have been observed in the absence of hypoxia [11]. Moreover, short waterlogging periods of 1–3 days can result in long-term detrimental effects on both the growth and yield of wheat [12]. Researchers believe that plants have established a set of new anatomical, morphological and physiological mechanisms to adapt to waterlogging environments [10], and these mechanisms include the production of aerenchyma, the formation of adventitious roots, petiole elongation, the growth of stem hypertrophy, the growth of hypocotyls, increases in plant height, stomatal closure, the reduction of transpiration and the inhibition of photosynthesis [2, 4, 13]. In the wheat research field, more attention has been paid to the response of plants to waterlogging at the seedling and filling stages [3, 14], but few studies have investigated the response of wheat to waterlogging stress at the germination stage.

Transcriptome sequencing technology can identify and clarify the metabolite synthesis pathways and can accelerate research on plant metabolism. RNA-Seq technology has been used to analyse the differential expression profile of waterlogging-tolerant and waterlogging-sensitive rice varieties during germination to rapidly screen several important candidate genes, such as *OsTPP7*, *OsHXK7* and *OsPGM*, which might affect the early stage of rice growth [15]. The germination of seeds is mainly dependent on amyloplasts in the endosperm [16]; therefore, we focused our analysis on the structural changes in the endosperm at the seed germination stage. In this study, the phenotype and anatomical structures of different wheat genotypes (waterlogging-tolerant, waterlogging-medium tolerant, and waterlogging-sensitive varieties) during seed germination were analysed, and the transcriptional profile during germination was then analysed. Comparisons of the structural and transcriptional profiles of three types of wheat under the waterlogging and control treatments revealed several key DEGs involved in important metabolic pathways under the waterlogging treatment. This study establishes a foundation for further study on the functional characteristics of key waterlogging resistance genes in wheat and is of great significance for the genetic improvement of waterlogging-resistant wheat varieties.

## Results

### Phenotypic analysis of different wheat varieties under the waterlogging treatment

**Fig. 1:**
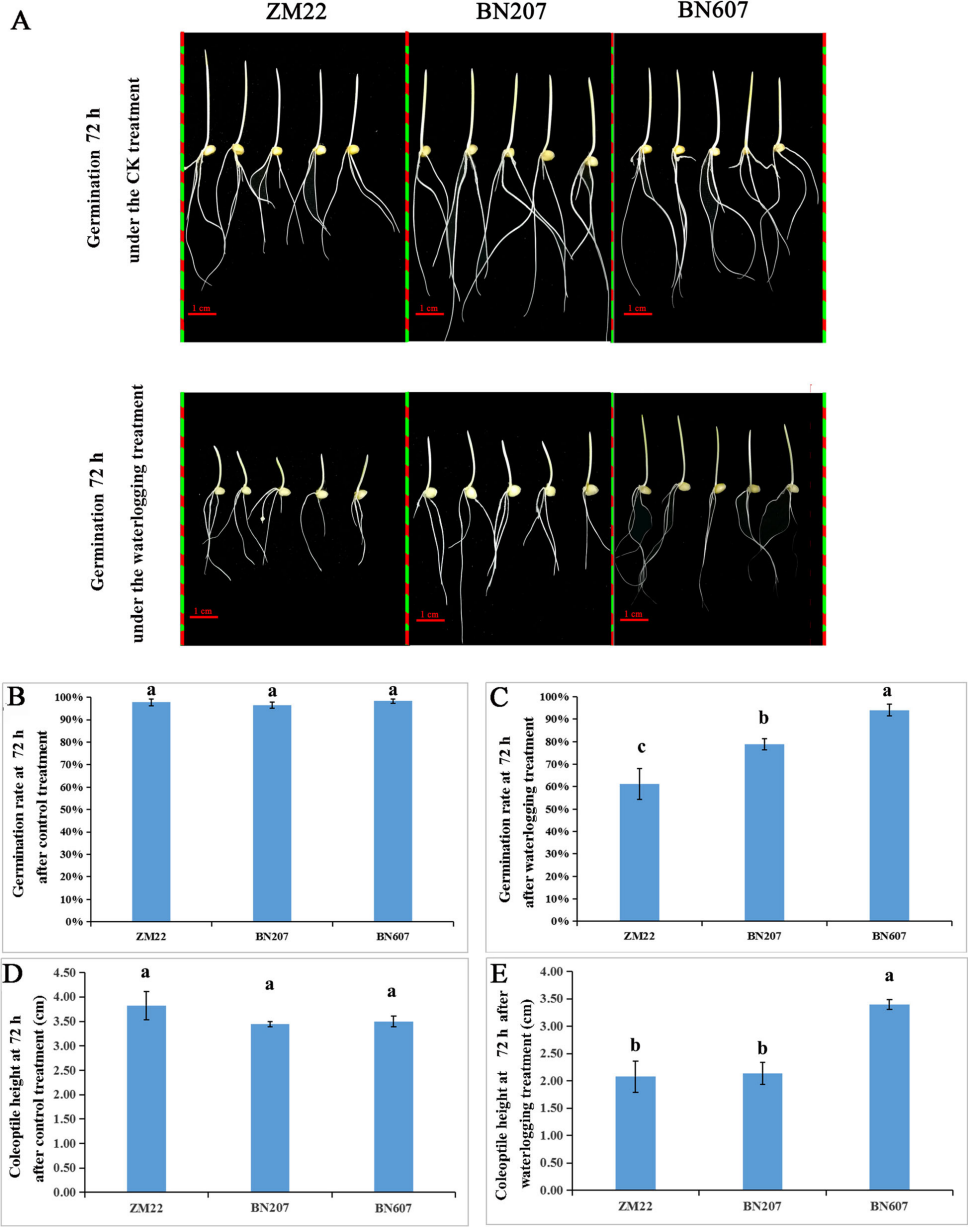
Phenotypic analysis of different wheat varieties after 72 h of germination under the control and waterlogging treatments. **a**, Phenotypes of ZM22, BN207 and BN607 seeds after 72 h of germination under the control and waterlogging treatments. Bars: 1 cm. **b**, Germination rate of ZM22, BN207 and BN607 after 72 h of the control treatment. **c**, Germination rate of ZM22, BN207 and BN607 after 72 h of the waterlogging treatment. **d**, Coleoptile height of ZM22, BN207 and BN607 after 72 h of the control treatment. **e**, Coleoptile height of ZM22, BN207 and BN607 after 72 h of the waterlogging treatment. Each point represents the average from five samples. The error bars represent the SDs. The statistical analysis was performed using the LSD test with *p* < 0.05

The phenotypes of three wheat seeds (ZM22, BN207 and BN607) after 3 days of germination under the waterlogging treatment are shown in Fig. 1. Under the control treatment, no significant difference in the coleoptile height (CH) was found among the three wheat varieties (Fig. 1a). The GR and CH of the three types of wheat were higher than 95% and approximately 3.5 cm, respectively (Fig. 1b and d). Although no significant differences were found among the roots of ZM22, BN207 and BN607, the GR and CH of the three wheat varieties under the waterlogging treatment were significantly different from those under the control treatment. The highest GR was found with BN607 (94.02%), followed by BN207 (78.85%), and the lowest GR was obtained with ZM22 (61.21%) (Fig. 1c and e). In addition, the CHs of ZM22 and BN207 under the waterlogging treatment (average height of approximately 2 cm) were significantly lower than those under the control treatment, whereas that of BN607 under the waterlogging treatment was equal to 3.5 cm and was not significantly different compared that under the control treatment (Fig. 1e). Based on the above analysis, we ranked the three wheat varieties based on their waterlogging tolerance levels of (from high to low) as BN607, BN207 and ZM22.

### Anatomical analysis of different wheat seeds under the waterlogging treatment

**Fig. 2:**
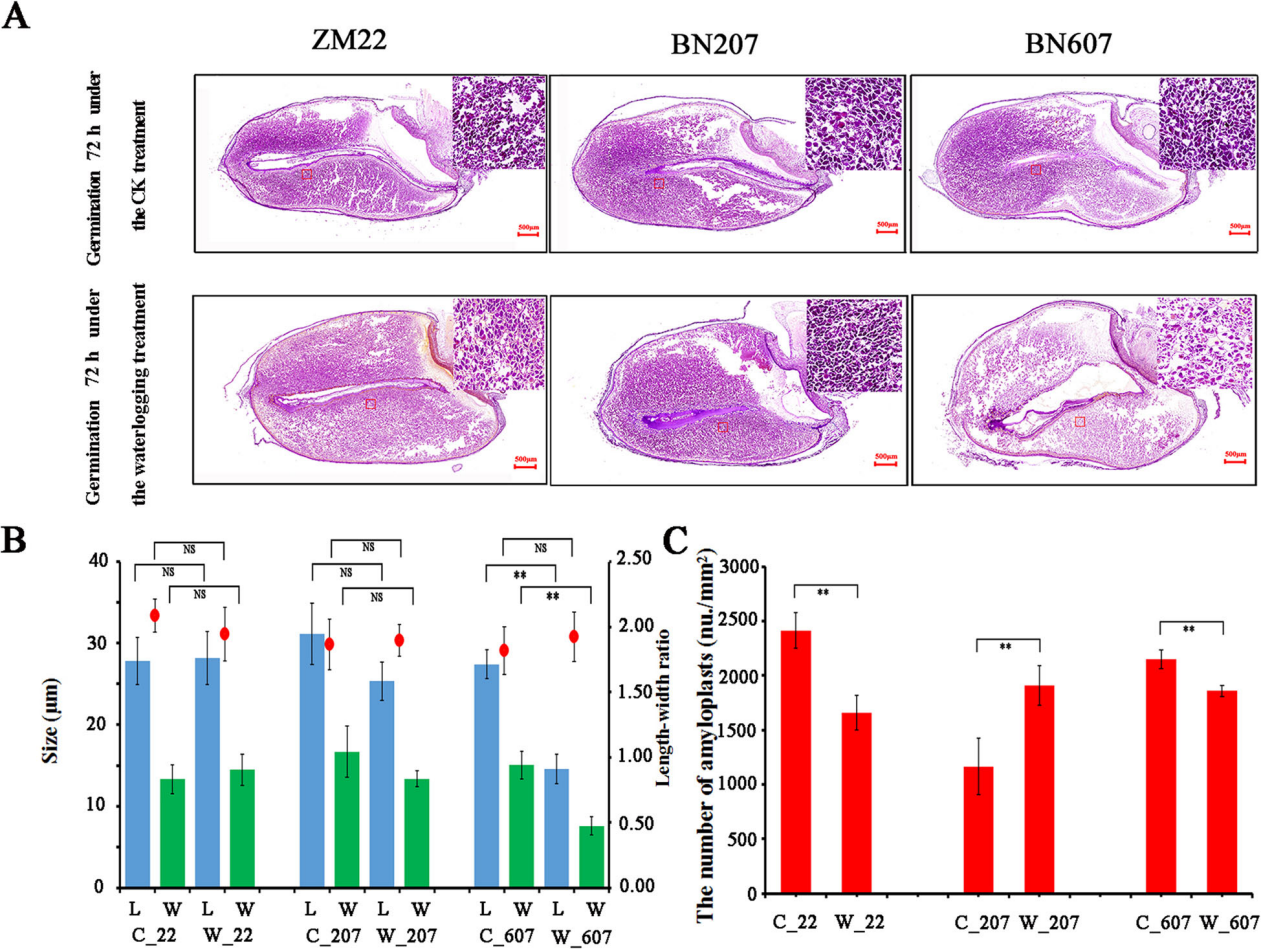
Structural anatomy of the endosperm of three wheat varieties after 72 h of germination under the control and waterlogging treatments. **a**, Structural anatomy of the endosperm of ZM22, BN207 and BN607 under the control and waterlogging treatments. Bars: 500 μm. **b**, Length, width and ratio of the length to the width of the amyloplasts of ZM22, BN207 and BN607. L, amyloplast length; W, amyloplast width. **c**, Number amyloplasts in ZM22, BN207 and BN607. ZM22: Zhoumai 22, BN207: Bainong 207, BN607: Bainong 607; C_22, ZM22 after 72 h of germination under the control treatment; W_22: ZM22 after 72 h of germination under the waterlogging treatment; C_207, BN207 after 72 h of germination under the control treatment; W_207, BN207 after 72 h of germination under the waterlogging treatment; C_607, BN607 after 72 h of germination under the control treatment; W_607, BN607 after 72 h of germination under the waterlogging treatment. Each point represents the average from three samples. The error bars represent the SDs. ∗ indicates significance at *p* < 0.05, ∗∗ indicates significance at *p* < 0.01

The structural anatomical analysis of the seeds showed that the endosperm was emulsified and degraded during seed germination. The endosperm began to emulsify at the area adjacent to the embryo, which resulted in the formation of a cavity (Fig. 2a). The emulsification degrees of the endosperms of the three wheat varieties after 72 h of waterlogging treatment were different. The highest degree of endosperm emulsification was obtained for BN607, followed by BN207 and ZM22 (Fig. 2a). As demonstrated by scanning slices of the three wheat varieties, no significant difference in the starch grain size of ZM22 between the waterlogging and control treatments, but the number of amyloplasts under the waterlogging treatment was significantly lower than that under the control treatment (Fig. 2b). In BN607, the length and width of the amyloplasts under the waterlogging treatment were lower than those under the control treatment, and the number of amyloplasts under the waterlogging treatment was significantly lower than that under the control treatment (Fig. 2c). In BN207, no significant difference in the starch size was detected between the waterlogging and control treatments. Surprisingly, the number of amyloplasts in ZM22 under the waterlogging treatment was significantly higher than that under the control treatment (Fig. 2c). We concluded that the amyloplasts of ZM22, BN207 and BN607 might exhibit varying levels of responses to waterlogging stress.

### Transcriptomic analysis of three wheat varieties under the waterlogging and control treatments

To study the response mechanism of wheat seeds to waterlogging stress during germination, samples were collected from BN607, BN207 and ZM22 under the waterlogging and control treatments. Each variety was subjected to three replicate treatments, and a total of 18 libraries were constructed. We subsequently performed transcriptome sequencing using Illumina NovaSeq™ 6000 with the wheat genome was used as the reference genome, and an average of 6.40 million reads were obtained from each sample. After removing low-mass, joint, and potentially contaminated data, 7.41–10.38 GB data were obtained from each sample, the GC value was 53.00–54.00%, and the Q30 value ranged from 97.62 to 98.44% (Table S1). The ratio of exons to reference genomic libraries averaged 92.35%, whereas the average ratios obtained for introns and intergenic spacers were 2.67 and 4.98%, respectively (Table S2).

A principal component analysis (PCA) of all the genes in the 18 libraries (Fig. S1) revealed that principal component 1 (PC1) and PC2 explained 79.09 and 11.85% of the variance. The three biological replicates of each treatment were significantly clustered together, and similar results were obtained with the waterlogging-treated and normally cultured wheat varieties. These results showed that the biological replicates of the samples were good and that the sequencing results were reliable.

### Analysis of DEGs in three wheat varieties in response to waterlogging stress

**Fig. 3:**
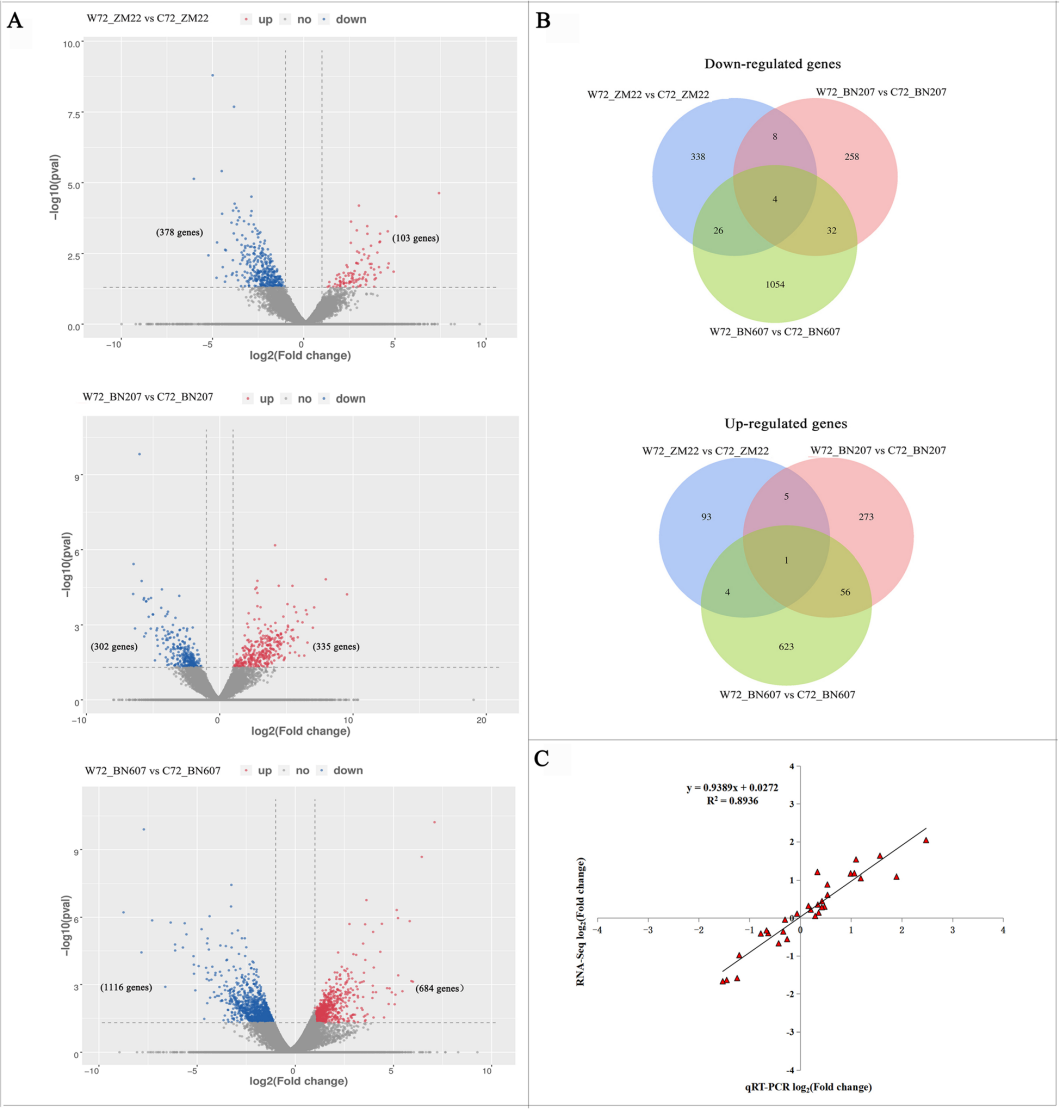
Analysis of DEGs in three wheat cultivars under waterlogging stress. **a**. Volcano plot analysis of DEGs in ZM22, BN206 and BN607. The abscissa represents the log_2_ (fold change), and -log_2_ (*p*-value) is shown as the ordinate. The red colour indicates significantly upregulated DEGs, the blue colour showed the significantly downregulated DEGs, and the grey colour represents nonsignificant DEGs. **b**. Venn diagram of downregulated and upregulated genes in ZM22, BN206 and BN607. **c**. Correlation analysis of the qRT-PCR data and the RNA sequencing results. W72_ZM22, W72_BN207 and W72_BN607 refer to ZM22, BN207 and BN607 under the waterlogging treatment, respectively. C72_ZM22, C72_BN207 and C72_BN607 refer to ZM22, BN207 and BN607 under the control treatment, respectively

A total of 107,891 genes were obtained from the transcriptome sequencing data, and 479, 637 and 1800 DEGs were identified in ZM22, BN207 and BN607 under waterlogging stress, respectively (Fig. 3a). As determined through a GO enrichment analysis of these DEGs (Fig. S2, Table S3), the DEGs in ZM22 mostly functioned in oxidoreductase activity and mainly participated in the biological response to abscisic acid. In addition, the DEGs in BN207 mainly functioned in molecular function and DNA binding, mostly exhibited protein heterodimerization activity and mainly participated in the biological response to abscisic acid, the response to water deprivation and the defence response to fungus. Moreover, the DEGs in BN607 mostly showed transcription factor activity and mainly participated in the biological oxidation-reduction process. A KEGG enrichment analysis showed that 13 DEGs in ZM22 were significantly enriched in the “amino sugar and nucleotide sugar metabolism” pathway (Fig. S3, Table S4), followed by the “glyceride metabolism” and “base sugar and nucleotide sugar metabolism” pathways. In BN607 under waterlogging stress, 40 genes were significantly enriched in the “phenylpropanoid biosynthesis” and “starch and sucrose metabolism” pathways followed by the “amino sugar and nucleotide sugar metabolism” pathway.

A total of 2775 genes were significantly expressed in three wheat varieties under waterlogging stress. In ZM22, 378 genes were significantly downregulated, and 103 genes were significantly upregulated (Fig. 3a), and 302 and 335 genes were significantly downregulated and significantly upregulated in BN207, respectively (Fig. 3a). In BN607, 1116 and 684 genes exhibited significantly downregulated expression and significantly upregulated expression, respectively (Fig. 3a). A Venn diagram of 2775 DEGs showed that 1677 DEGs were uniquely found in BN607, and 1054 and 623 genes were downregulated and upregulated, respectively. In addition, five DEGs were shared among the three varieties. The expression difference levels of four genes (*TraesCSU02G032900*, *MSTRG.18850*, *TraesCS6D02G038500* and *TraesCS1A02G139900*) were downregulated in the three varieties under waterlogging stress, and the expression level of *TraesCS5B02G445500* was upregulated under waterlogging stress (Fig. 3b, Table S5), which suggested that these genes might play an important role in three wheat varieties under waterlogging stress.

To validate the reliability of the expression profiles obtained by RNA-Seq, we randomly selected 10 DEGs that showed various expression levels for validation by quantitative real-time polymerase chain reaction (qRT-PCR). Pearson’s correlation coefficients showed that the qRT-PCR and RNA sequencing data for these genes were highly correlated (Fig. 3c). The correlation coefficient was 0.8936, which indicated a positive correlation between the RNA sequencing and qRT-PCR data (Fig. 3c).

### Selection of gene set response to waterlogging stress by hierarchical clustering based on DEGs

**Fig. 4:**
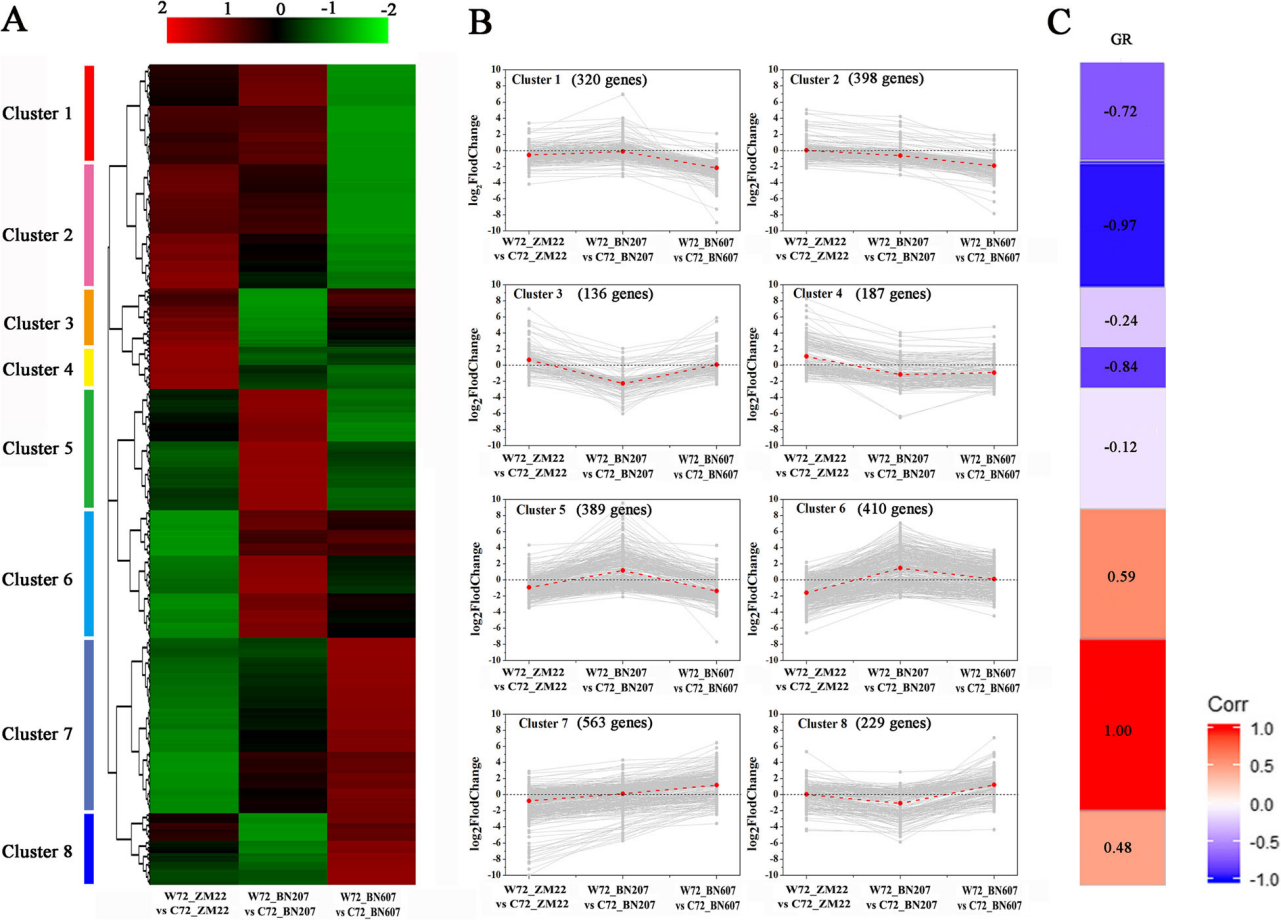
Cluster analysis of DEGs in ZM22, BN207 and BN607 under waterlogging stress. **a**. The DEGs with similar expression patterns were grouped into the same cluster. **b**. The ordinate indicates the relative expression of DEGs, standardized by log_2_ (fold change), and the abscissa indicates ZM22, BN207 and BN607. **c**. Correlation analysis between the FPKM values of all DEGs in the eight clusters and the germination percentage obtained for the three wheat cultivars under the waterlogging treatment. The colour of the heat map from top to bottom corresponds to Clusters 1 to 8. GR: germination rate. W72_ZM22, W72_BN207 and W72_BN607 refer to ZM22, BN207 and BN607 under the waterlogging treatment, respectively. C72_ZM22, C72_BN207 and C72_BN607 refer to ZM22, BN207 and BN607 under the control treatment, respectively

According to the differences in the expression of the DEGs among the three waterlogging-resistant varieties (BN607 > BN207 > ZM22), the DEGs were stratified and clustered (Fig. 4a). The results showed that these DEGs were divided into eight clusters with different expression patterns (clusters 1 to 8, Fig. 4b, Table S7). Among these clusters, cluster 7 contained 563 DEGs, and the expression of these DEGs in the three varieties was positively correlated with the GR (R^2^ = 1.00). In contrast, cluster 2 contained 398 DEGs, and the expression of these genes in the three varieties was negatively correlated with the GR (R^2^ = -0.97, Fig. 4c). The results from the above analysis suggested that the DEGs in clusters 2 and 7 play important roles in the response of the three wheat cultivars to waterlogging stress.

### Main metabolic pathways involved in wheat germination under waterlogging stress

Based on the hierarchical clustering analysis of the DEGs, we focused on analysing the DEGs in clusters 2 and 7 that are involved in waterlogging stress. GO and KEGG enrichment analyses revealed that these genes were mainly involved in the glycolysis pathway, the starch and sucrose metabolism pathway and the lactose metabolism pathway.

### Analysis of DEGs related to the glycolytic pathway

**Fig. 5:**
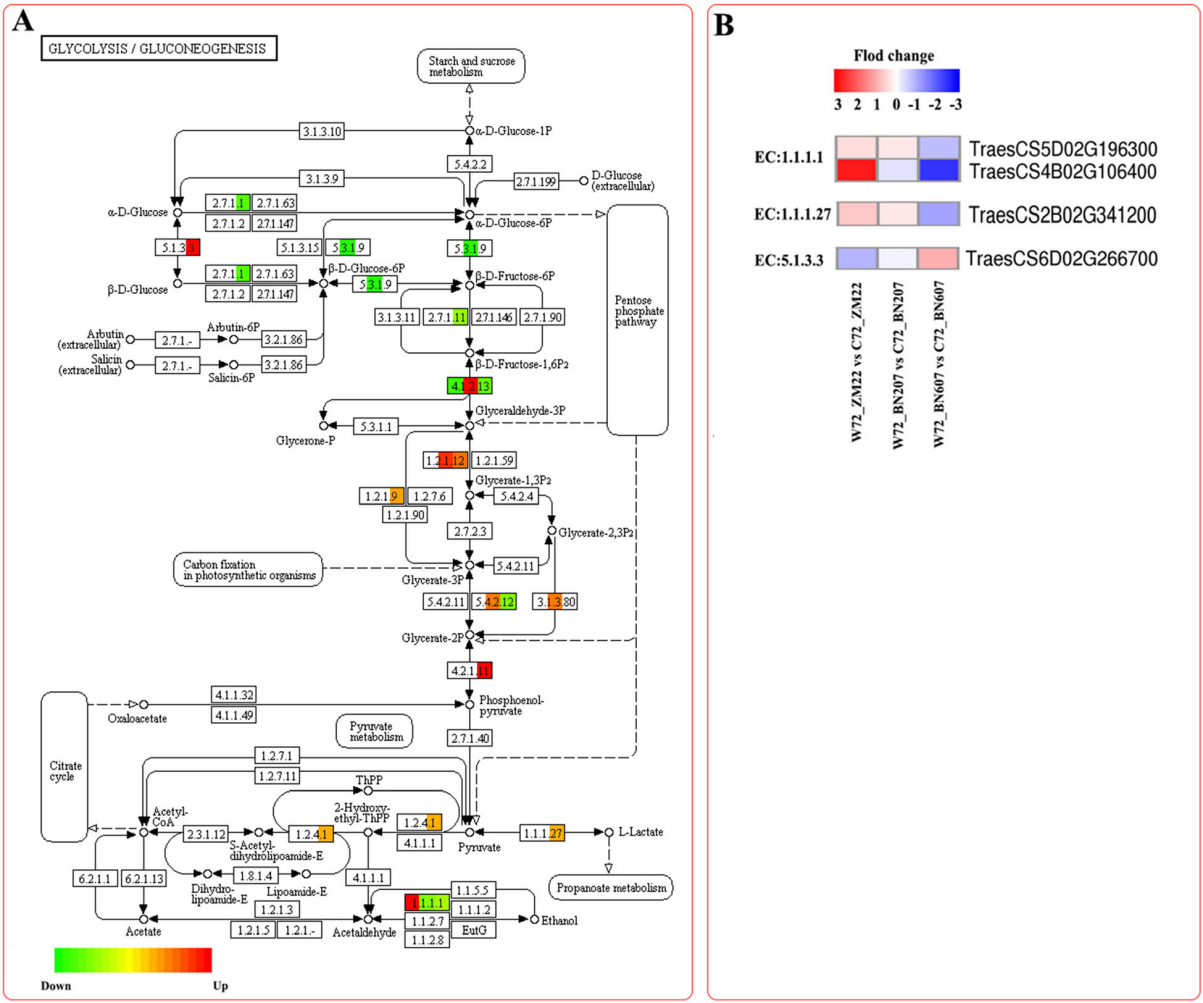
Analysis of DEGs involved in the glycolysis pathway in ZM22, BN207 and BN607. **a**, Pattern diagram of the glycolysis pathway. The red squares indicate upregulated genes, the green squares indicate downregulated genes, and the orange squares indicate both upregulated and downregulated genes. **b**, The expression difference levels of DEGs involved in the glycolysis pathway between waterlogging treatment and control treatment in three varieties. The metabolism of each previously selected DEGs was analysed using a heatmap. Relative expression level of DEGs was measured by log2 ratio (Fold change between waterlogging treatment and control treatment each variety). Positive fold change values (red) indicate the up-regulation, whereas negative fold change values (blue)

The KEGG enrichment analysis showed that many DEGs in the three types of wheat under the waterlogging and control treatments were involved in the glycolysis pathway (Fig. 5a). We screened four DEGs in clusters 2 and 7 (two ADH genes, one L-lactate dehydrogenase gene, and one glycosyltransferase gene) that were closely related to the GR (Fig. 5b). The expression difference levels of two genes (*TraesCS5D02G196300* and *TraesCS4B02G106400*) encoding alcohol dehydrogenase decreased successively in ZM22, BN206 and BN607 (Fig. 5b). In addition, the expression trend of the L-lactate dehydrogenase gene (*TraesCS2B02G341200*), which catalyses the conversion of pyruvate to L-lactate (EC: 1.1.1.27), was opposite to that found for the ADH gene in the three wheat varieties. Glycosyltransferase catalyses the conversion of α-D-glucose into β-D-glucose (EC: 5.1.3.3), and the expression difference levels of the gene (*TraesCS2B02G341200*) encoding this function decreased successively in ZM22, BN206 and BN607.

### Analysis of DEGs related to the starch and sucrose metabolism pathway

**Fig. 6:**
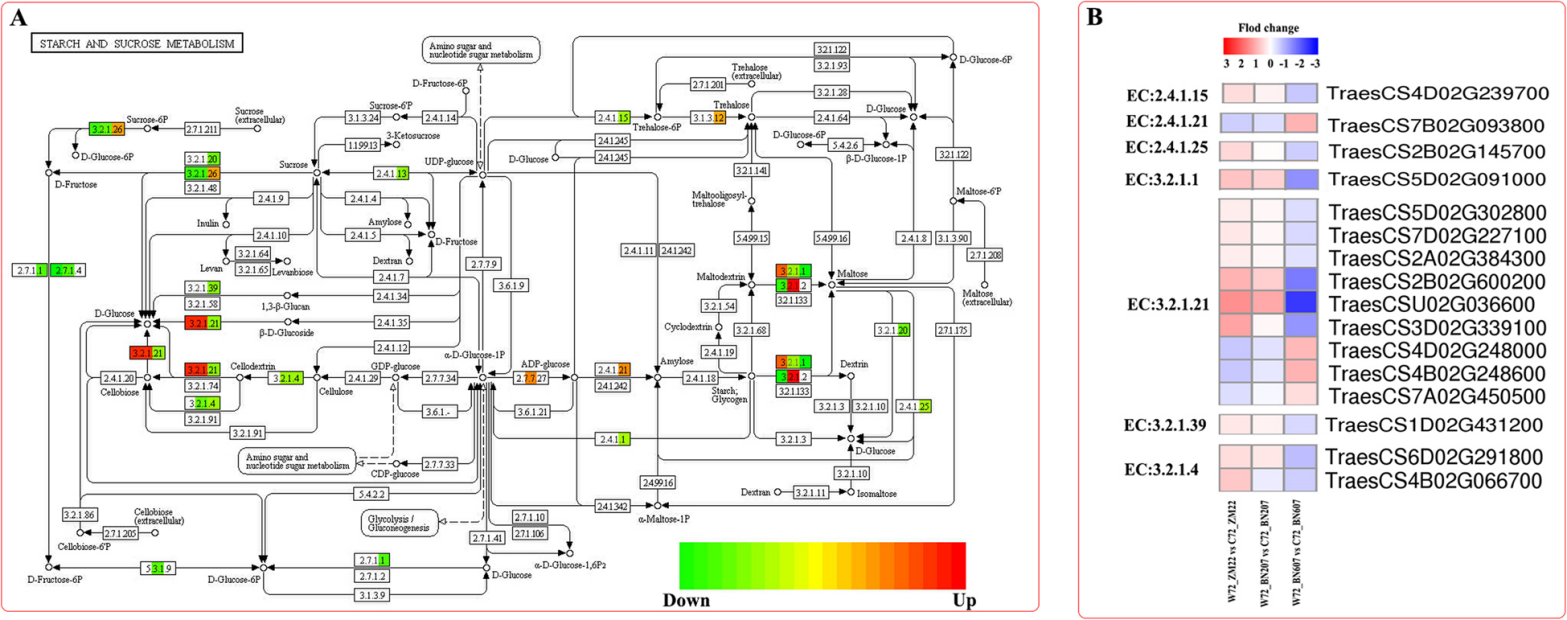
Analysis of DEGs involved in the starch and sucrose metabolic pathway in ZM22, BN207 and BN607. **a**, Pattern diagram of the starch and sucrose metabolic pathway. The red squares indicate upregulated genes, the green squares indicate downregulated genes, and the orange squares indicate both upregulated and downregulated genes. **b**, The expression difference levels of DEGs involved in the starch and sucrose metabolic pathway between waterlogging treatment and control treatment in three varieties. The metabolism of each previously selected DEGs was analysed using a heatmap. Relative expression level of DEGs was measured by log2 ratio (Fold change between waterlogging treatment and control treatment each variety). Positive fold change values (red) indicate the up-regulation, whereas negative fold change values (blue)

The KEGG enrichment revealed that a large number of the DEGs in the three wheat varieties under the waterlogging and control treatments were involved in the starch and sucrose metabolism pathway (Fig. 6a), and 18 of the DEGs in clusters 2 and 7 that were involved in the starch and sucrose metabolism pathway were screened (Fig. 6b). A gene (*TraesCS5B02G015800*) belonging to cluster 7 was annotated as alpha-amylase, and the expression difference levels increased successively in ZM22, BN206 and BN607, which indicated that this gene might play an important role in starch decomposition. We screened the gene (*TraesCS7B02G093800*) encoding maltitol glycosylase (EC: 2.4.1.21), and these expression difference levels successively increased in ZM22, BN206 and BN607. In addition, the expression difference levels of *TraesCS5D02G09100*, which encodes the enzyme that catalyses the conversion of starch/glycogen to dextrin, was decreased successively in ZM22, BN206 and BN607. This result indicated that the ability of ADP-glucose to control amylose synthesis was gradually enhanced. Chitinase was used to catalyse the decomposition of *β*-D-glucose or fibre to D-glucose (EC: 3.2.1.21). The expression difference levels of six genes encoding this function decreased gradually in ZM22, BN206 and BN607, but the opposite trend was found for three other genes (Fig. 6b). In this study, the expression difference levels of the genes encoding glucan-1,3-glucosidase (EC: 3.2.1.39) and endocellulase (EC: 3.2.1.4) decreased gradually in ZM22, BN206 and BN607 (Fig. 6b), which indicated that these genes might play an important role in the waterlogging tolerance of different varieties.

### Analysis of DEGs related to the lactose metabolic pathway

**Fig. 7:**
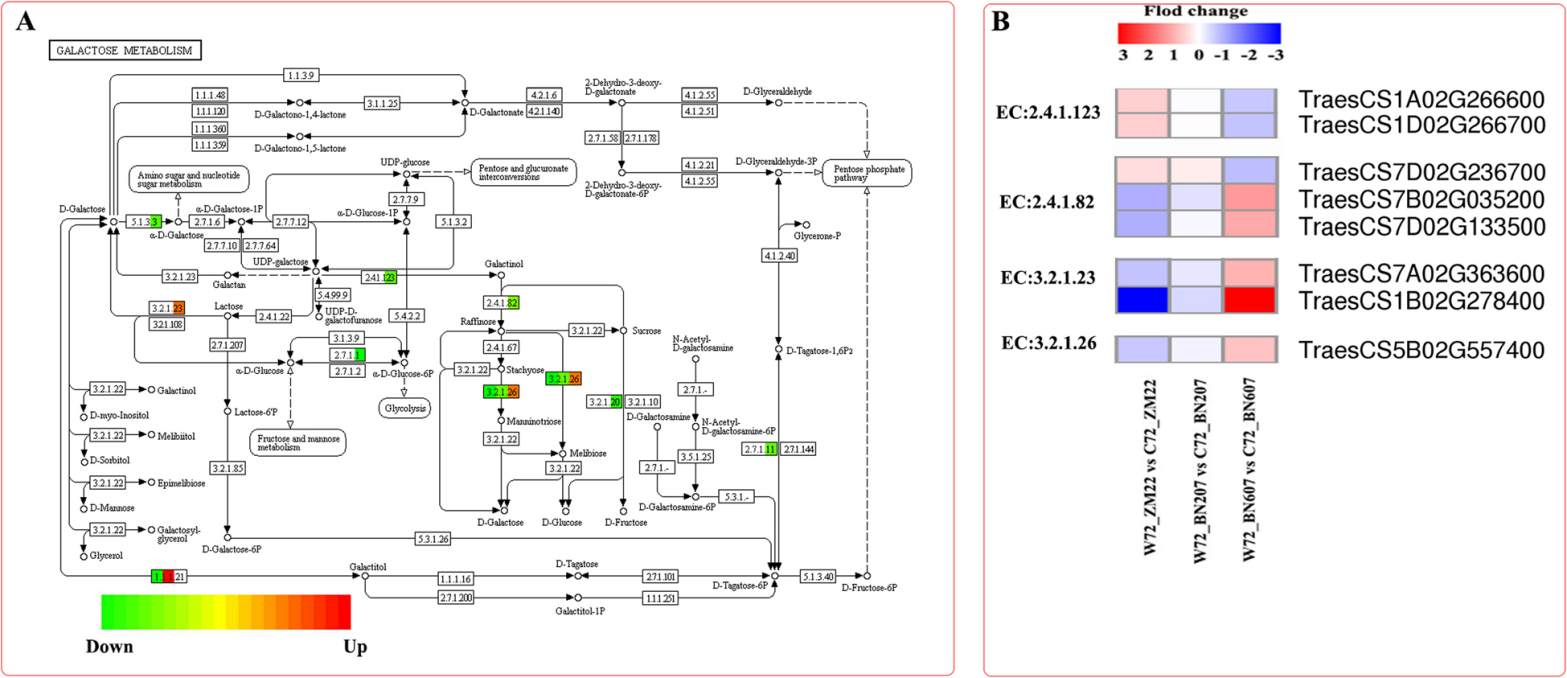
Analysis of DEGs involved in the lactose metabolic pathway in ZM22, BN207 and BN607. **a**, Pattern diagram of the lactose metabolic pathway. The red squares indicate upregulated genes, the green squares indicate downregulated genes, and the orange squares indicate both upregulated and downregulated genes. **b**, The expression difference levels of DEGs involved in the lactose metabolic pathway between waterlogging treatment and control treatment in three varieties. The metabolism of each previously selected DEG was analysed using a heatmap. Relative expression level of DEGs was measured by log2 ratio (Fold change between waterlogging treatment and control treatment each variety). Positive fold change values (red) indicate the up-regulation, whereas negative fold change values (blue)

A comprehensive analysis of the DEGs in the three wheat varieties belonging to clusters 2 and 7 and involved in the lactose metabolism pathway of (Fig. 7a) identified two genes (*TraesCS1A02G266600* and *TraesCS1D02G266700*) that encode glucuronosyltransferase (inositol galactoside synthase) (EC: 2.4.1.123). This protein catalyses the synthesis of inositol galactoside from UDP-galactose and myoinositol, which is the first key step in the synthesis of raffinose family oligosaccharides (RFOs) and the most critical regulatory step in RFO synthesis. The expression difference levels of these two genes were decreased successively in ZM22, BN206 and BN607. The analysis identified three genes encoding inositol galactoside-sucrose galactosyltransferase (raffinose synthase) (EC: 2.4.1.82). This protein mainly catalyses the synthesis of raffinose from inositol galactoside and sucrose. The expression difference levels of the *TraesCS7D02G236700* gene were gradually decreased in ZM22, BN206 and BN607, whereas those of the *TraesCS7B02G035200* and *TraesCS7D02G133500* genes were gradually increased in ZM22, BN206 and BN607. One gene (*TraesCS5B02G557400*) encoding *β*-D-fructofuranoside (EC:3.2.1.26), which mainly catalyses the decomposition of stachyose and raffinose into melibiose and the decomposition of sucrose into glucose and fructose, was identified. The expression difference levels of *TraesCS5B02G557400* were gradually increased in ZM22, BN206 and BN607. *β*-galactosidase (EC:3.2.1.23) mainly catalyses the decomposition of lactose and raffinose into melibiose. We also found that the expression difference levels of two genes (*TraesCS7A02G0363600* and *TraesCS1B02G278400*) gradually increased in ZM22, BN206 and BN607.

### Analysis of DEGs related to transcription factors

The analysis of the DEGs in clusters 2 and 7 revealed 23 transcription factors involved in waterlogging stress (Table 1). These transcription factors were distributed in 12 families, including the WRKY, MYB, MADS, ZIP, and ERF families (Table 1). Among the WRKY family, the expression difference levels of five genes was highest in ZM22, followed by BN207 and then BN607, whereas the expression difference level of one gene was lowest in ZM22, followed by BN207 and then BN607. A total of four genes related to the MYB family were found: The expression difference levels of one of these genes gradually decreased in ZM22, BN206 and BN607, and those of the other three genes gradually increased. In addition, the expression difference levels of *MSTRG.6000*, *TraesCS1B02G392300* and *MSTRG.32294*, which are all ethylene-related transcription factors (ERF, AP2/ERF, and ABR) belonging to cluster 7, gradually increased in ZM22, BN206 and BN607. This finding indicated that these transcription factors might play an important role in the response to waterlogging stress in different wheat varieties.

**Table 1:** Response of transcription factors to waterlogging stress

Category	Gene_id	Cluster	Log_2_ (Fold change)			Swissprot_name	Swissprot_description
			W72_ZM22 vs C72_ZM22	W72_BN207 vs C72_BN207	W72_BN607 vs C72_BN607		
WRKY	TraesCS1A02G197400	C2	−0.13	−0.38	−1.55	WRKY35	WRKY transcription factor 35
	TraesCS3A02G347500	C2	−0.4	− 0.57	−1.83	WRKY24	WRKY transcription factor WRKY24
	TraesCS6A02G146900	C2	−0.99	−1.07	− 2.16	WRKY71	WRKY transcription factor WRKY71
	TraesCS6B02G175100	C2	−0.98	−1.4	− 2.14	WRKY71	WRKY transcription factor WRKY71
	TraesCS2B02G410400	C2	−0.13	− 0.61	−1.51	WRKY12	WRKY transcription factor 12
	TraesCS3D02G113300	C7	−2.2	−0.49	0.15	WRKY72	WRKY transcription factor 72
MYB	TraesCS1D02G084400	C2	0.04	−0.65	−1.68	MYB86	Transcription factor MYB86
	TraesCS7D02G514800	C7	−3.69	−1.34	−0.03	MYB44	Transcription factor MYB44
	TraesCS2A02G157600	C7	−1.46	−0.46	0.19	MYB306	Myb-related protein 306
	TraesCS2A02G116100	C7	0.7	0.78	1.03	EFM	Myb family transcription factor EFM
ERF	TraesCS2A02G288000	C7	−0.06	0.32	1.12	ERF114	Ethylene-responsive transcription factor ERF114
	TraesCS1B02G392300	C7	−0.41	0.73	1.95	AP2/ERF	AP2/ERF and B3 domain-containing protein
	TraesCS7A02G264100	C7	−0.2	0.39	1.08	ABR1	Ethylene-responsive transcription factor ABR1
MADS-box	TraesCS5B02G115100	C2	0.17	0.06	−2.07	MADS13	MADS-box transcription factor 13
	TraesCS4D02G346300	C7	0.07	0.96	1.16	MADS51	MADS-box transcription factor 51
ZIP	TraesCS2A02G424200	C2	0.14	−0.34	−1.26	ZIP3	Zinc transporter 3
	TraesCS1B02G306500	C2	−0.14	−0.34	−1.36	ZIP5	Zinc transporter 5
BHLH	TraesCS2D02G347200	C7	−1.1	−0.56	1.62	BHLH113	Transcription factor bHLH113
	TraesCS5D02G411600	C7	−2.61	0.24	1.16	BHLH84	Transcription factor bHLH84
IAA	TraesCS7A02G322000	C2	−0.74	−0.99	−1.55	IAA25	Auxin-responsive protein IAA25
ARF	TraesCS3B02G190100	C2	−0.1	−0.64	−1.34	ARF1	Auxin response factor 1
HSFA	TraesCS2D02G211400	C7	−1.72	−0.81	0.53	HSFA3	Heat stress transcription factor A-3
NAC	TraesCS2A02G102000	C7	0.24	0.69	1.24	NAM-B1	NAC transcription factor NAM-B1

NOTE: C72_ZM22, ZM22 after 72 h of germination under the control treatment; W72_ZM22: ZM22 after 72 h of germination under the waterlogging treatment; C72_BN207, BN207 after 72 h of germination under the control treatment; W72_BN207, BN207 after 72 h of germination under the waterlogging treatment; C72_BN607, BN607 after 72 h of germination under the control treatment; and W72_BN607, BN607 after 72 h of germination under the waterlogging treatment

## Discussion

### Response of the GR of different wheat varieties to waterlogging stress

Waterlogging often occurs at all stages of plant growth; therefore, evaluating the waterlogging tolerance of plants at each stage is highly important. Studies of the physiological and molecular mechanisms related to the effects of waterlogging in wheat at the seedling stage [9, 14], filling stage [17] and maturity stage [18] have been reported. Some studies have revealed that wheat, barley and rape are markedly more likely to suffer from waterlogging within 2 and 6 weeks after germination than between 6 and 14 weeks after germination. In addition, Cannell et al. [19] found that after wheat germination, all seedlings died after 16 days of waterlogging at 12 °C, whereas the survival rate of wheat seedlings after 6 days of waterlogging was 12–38% of that found with the control treatment. Considerable differences in waterlogging tolerance have been found among different varieties in different regions. Takeda and Fukuyama [20] found that some varieties from China, Japan, South Korea and Nepal, as well as some varieties from North Africa, Ethiopia and southwestern Asia, tend to exhibit higher “waterlogging tolerance”, whereas barley varieties from western India show weaker “waterlogging tolerance” [8]. The plant height, soil and plant analyser development (SPAD) results, number of tillers, and biomasses of the stems and roots of non-waterlogging wheat varieties are lower than those of waterlogging-tolerant wheat varieties [21]. This study revealed that the GRs of ZM22, BN207 and BN607 increased gradually during waterlogging treatment. The CH and root length of ZM22 were seriously inhibited by waterlogging treatment for 72 h, whereas the growth of BN607 was not affected by the waterlogging treatment (Figs. 1 and 3), which indicated the existence of notable differences in the “waterlogging tolerance” among different varieties. The number of DEGs that were both up-regulated and down-regulated in the three varieties was very small (Fig. 3b). On the one hand, it may be related to the sampling period after the waterlogging treatment. This study only analyzed the period when the three varieties had significant differences in GR. For samples, studies have shown that significant changes have taken place inside the seeds when the seeds have been submerged for a few hours [8]. On the other hand, the speed of the physical and chemical changes in the embryo and endosperm of the three varieties of wheat seeds after waterlogging treatment is not consistent. Therefore, when sampling at a certain time point, there will be a phenomenon that the number of genes that were both up-regulated and down-regulated was small. However, it could be observed in Fig. 4 that there were many DEGs in the three varieties after waterlogging treatment. The focus of this study is to screen for DEGs that have a significant positive and negative correlation with GR. The expression of DEGs respond to waterlogging treatments had a significant effect on GR, which provides us with an important resource to explain the different mechanisms of submergence tolerance of different wheat varieties and to study key candidate genes that regulate seed submergence tolerance during wheat germination.

### Anatomy of the grain endosperm and endosperm in the response of wheat to waterlogging stress

The endosperm is the main location of nutrient storage in cereal seeds and mainly contains starch. After degradation by amylase, starch is transported to the germ and radicle in the form of simple compounds (such as sucrose), provides a matrix for respiration and serves as a material basis and energy source for radicle and germ growth and organ formation [16, 22, 23]. At the initial stage of wheat seed germination, the cell wall of endosperm cells is dissolved, and the endosperm cells are separated. The starch and cellulose gradually decompose under a series of enzymes (α−/β-amylase and cellulases), which loosens the entire endosperm structure and leads to endosperm liquefaction. Therefore, the activity of amylase in seed germination might be determined by the degrees of endosperm emulsification and degradation. This study revealed that the endosperm cells of three types of wheat seeds were markedly changed in response to waterlogging stress. Specifically, the degree of emulsification between the embryo and the endosperm found for BN207 and BN607 was higher than that obtained for ZM22, and BN607 exhibited the highest degree of emulsification (Fig. 2b and c). The differences in the number and size of amyloplasts among ZM22, BN2607 and BN607 might be due to the inconsistencies in the degradation rate. For example, compared with the control treatment, the size of amyloplasts in ZM22 remained unchanged, but the number of amyloplasts was decreased by the waterlogging treatment. In addition, the size of amyloplasts in BN207 was decreased under the waterlogging treatment, but the difference between the control and waterlogging treatments was not significantly. Moreover, the number of amyloplasts in BN207 increased, and starch degradation was rapidly completed, which provided nutrients for embryo growth.

### Importance of the ADH gene in the response of wheat to waterlogging stress at the germination stage

Seed germination requires an energy supply, and energy metabolism is activated only when the seed is imbibed. Studies have revealed that some proteins related to energy metabolism are phosphorylated in wheat embryos. For example, the upregulation of the expression of the *ADH* gene under waterlogging stress servers as a positive signal. Alcohol fermentation in plants is activated under low oxygen stress conditions: pyruvate decarboxylase (PDC) first converts pyruvate to acetaldehyde, and alcohol dehydrogenase then convert acetaldehyde to ethanol. It has been reported that at the early stage of soybean growth and development, the expression of *ADH* and *PDC* in soybean roots is significantly upregulated under waterlogging stress [24–26]. It has also been found that hypoxia stress can lead to the production of fermentation alcohol products [PDC, lactate dehydrogenase (LDH), and ADH] in *Arabidopsis thaliana* [27]. Komatsu et al. [28] reported that there are at least six *ADH* genes involved in the response of soybean to waterlogging stress, and one of these genes, *GmADH2*, is specifically expressed in soybean root tissue. Similarly, two *ADH* genes have been cloned in cotton, and only one of these genes is expressed under waterlogging stress [29]. Zhang et al. [30] found that two of the three *ADH* genes in kiwifruit were significantly upregulated in the roots after waterlogging treatment. Tougou et al. [26] found that the inhibitory effect of waterlogging stress on the growth of soybean seedlings was decreased in *GmADH2I-*overexpressing transgenic soybeans, whereas the expression and activity of ADH in the transgenic soybeans were higher than those in the control soybeans, which suggested that the exposure of *GmADH2* transgenic soybeans to waterlogging stress might induce changes in glycolysis and alcohol fermentation and improve the GR. The transcriptional levels of metabolites in the embryo and endosperm of rice seeds are increased by anaerobic treatment for 12 h, and the greatest change in a large number of metabolites has been observed after 48 h of treatment [31]. This finding shows that the enzyme activity-catalysed energy metabolism is induced or activated at the beginning of germination, and the late changes in metabolites might be driven by transcription and translation because these occurred after the observed changes in the transcriptional abundance. In this study, we found that the transcriptional levels of *ADH2* (*TraesCS5D02G196300*) and *ADH3* (*TraesCS4B02G106400*) (Fig. 7) after 72 h of the waterlogging treatment were lower than those obtained with the control treatment, and the greatest decrease was observed in ZM22, followed by BN207 and then BN607 (data not shown). In contrast, the transcriptional levels of these genes increased gradually after 1 day of the waterlogging treatment (data not shown). This finding suggested that the *ADH* genes in the endosperm of BN607 were induced rapidly after short-term waterlogging, and the glycolysis pathway provided the energy needed to induce the early germination of BN607 seeds. However, the *ADH* genes of ZM22 were highly expressed after 72 h of the waterlogging treatment. It is possible that the accumulation of toxic substances, such as acetaldehyde, at the early stage inhibits seed germination.

### Waterlogging affects sugar metabolism of endosperm and endosperm in wheat

The energy metabolism pathway is not only related to the alcohol dehydrogenase-catalysed step of glycolysis but also associated with starch and sucrose metabolism and the lactose metabolism pathway [32, 33]. We found that after 72 h of waterlogging treatment, the expression levels of genes related to glycolysis, starch and sucrose metabolism and lactose metabolism in the embryo and endosperm of BN607 seeds were lower than those in ZM22 seeds. Among these genes, the expression difference levels of genes related to hydrolytic enzymes, such as chitinase, glucan-1,3-glucosidase and endocellulase, decreased successively in ZM22, BN206 and BN607. The trend underlying the changes in these genes was largely consistent with that found for *ADH* gene expression. The anatomical structure of the three wheat species and the expression levels of the analysed genes indicated that the emulsification and dissolution of the endosperm of BN607 seeds began early after waterlogging treatment, whereas the emulsification and dissolution of the embryo and endosperm of ZM22 seeds occurred relatively late. The transcriptional expression of mRNAs in ZM22 seeds was inhibited by waterlogging stress, and this inhibition occurred too late for the plant to switch to using starch and sugar hydrolysis to provide the energy needed for coleoptile growth.

The coleoptile is the protective tissue of young cotyledons of crops. The length and elongation rate of the coleoptile affect the early growth of crops. Coleoptile growth is considered the extension growth of coleoptile cells, and the power for this extension growth originates from continuous swelling and pressure. The extended growth of cells is limited by the cell wall; therefore, relaxation of the cell wall is necessary during coleoptile growth [34]. The relaxation of the cell wall is related to the degradation of hemicellulose, and a single hydrolase, such as β-glucanase, expansin, α-amylase and β-galactosidase, can induce cell wall relaxation. The pectin polysaccharides in the cell wall are hydrolysed by β-galactosidase, and this process is positively correlated with cell wall relaxation [35]. This study also found that the CH of ZM22, BN207 and BN607 and the expression levels of the two genes encoding β-galactosidase were increased by 72 h of the waterlogging treatment (Fig. 7b). We also found that the expression difference levels of a gene encoding α-amylase in cluster 7 also increased successively in ZM22, BN206 and BN607. This finding showed that the elongation of the wheat coleoptile under waterlogging stress might be closely related to the changes in the cell wall composition and the synthesis and activity of induced enzymes related to cell wall relaxation.

### Role of transcription factors in the response to waterlogging stress

Previous studies have found that WRKY-type genes play an important role in the responses to external abiotic stresses, such as drought, high salinity, cold injury, and heat damage [36], and some studies have also investigated WRKY-type genes in response to waterlogging stress. Nanjo et al. [25] found that the expression levels of WRKY-type transcription factors in soybean roots and hypocotyls are significantly upregulated under waterlogging treatment, and the expression levels of 11 WRKY transcription factor genes in the roots of kiwi fruit seedlings are significantly upregulated under waterlogging treatment [30]. This study revealed that the expression difference levels of six WRKY transcription factors gradually decreased in ZM22, BN206 and BN607 (Table 1), which indicated that the germination of ZM22, BN207 and BN607 seeds under the waterlogging treatment was negatively regulated by WRKY transcription factors. AP2/ERF plays an important role in the responses to external biotic and abiotic stresses [37–39]. SUB1A, an ERF transcription factor, can inhibit ethylene synthesis and the response of rice to gibberellin under waterlogging stress, reduce carbohydrate consumption and improve the waterlogging tolerance [40, 41]. Lee et al. [42] found that *CIPK15* expression is induced by hypoxia and a lack of glucose during rice germination under waterlogging, which causes a series of potential downstream chain reactions by activating the energy and stress receptive factor SnRK1. This effect can enhance the expression of amylolytic enzyme genes and the synthesis of alcohol dehydrogenase, which would promote anaerobic respiration to ensure the provision of sufficient metabolic energy for cell growth. During this time, the coleoptile can also undergo rapid elongation. In this study, the expression difference levels of one AP2/ERF transcription factor (*TraesCS1B02G392300*) increased successively in ZM22, BN206 and BN607 (Table 1), which indicated that the expression level of the AP2/ERF transcription factor could be used as an important reference index for evaluating the difference in waterlogging tolerance among the three wheat varieties. Further research on the regulatory mechanism of AP2/ERF transcription factors is needed in the future.

## Conclusions

In this study, three wheat varieties with different waterlogging tolerances were subjected to waterlogging treatment. Analyses of the seed GRs and the changes in the embryo and endosperm structures revealed that BN607 exhibited the strongest waterlogging tolerance, followed by BN207 and ZM22. To further reveal the difference in waterlogging tolerance among the three wheat varieties during germination, a transcriptome analysis was performed, and the results identified a total of 2775 DEGs between the control and waterlogging treatments. Based on the correlations between the DEGs and the GR, 563 and 398 key genes that exhibited positive and negative correlations, respectively, were screened. GO and KEGG analyses showed that the waterlogging tolerance of the three wheat varieties was closely related to glycolysis, starch and sucrose metabolism and the lactose metabolism pathway and that ADH genes played an important role in the response to short-term waterlogging. These results will provide insights into the mechanistic regulation of some candidate genes and could thus reveal the molecular mechanisms underlying the waterlogging tolerance of wheat seeds during the germination stage.

## Methods

### Experimental materials and treatment methods

In this study, three wheat (*Triticum aestivum* L.) varieties, ‘Zhoumai 22’ (ZM22, waterlogging-intolerant), ‘Bainong 207’ (BN207, waterlogging-medium) and ‘Bainong 607’ (BN607, waterlogging-tolerant), were selected as the materials. ZM22 is a commercial winter wheat cultivar in Henan Province, China. The BN207 and BN607 seeds used in this study were cultivated by Prof. Xingqi Ou from the School of Life Science and Technology, Henan Institute of Science and Technology, Xinxiang, China. All the seeds were provided by the School of Life Science and Technology, Henan Institute of Science and Technology. Intact seeds with uniform sizes and no pests or diseases were selected for the experiment. The seeds were first disinfected with 5% hydrogen peroxide for 5 min, washed three to five times with steam water, and submerged 12-cm-diameter glass Petri dishes filled with 150 mL of sterilized deionized water, and the Petri dishes were covered with aluminium foil to minimize gas exchange.

Experimental treatment: For the waterlogging treatment, 50 plump seeds in glass Petri dishes were incubated at a constant temperature of 20 °C in the dark for 72 h. At the end of the waterlogging treatment, the seeds were transferred and germinated in a Petri dish containing one layer of filter paper and 10 mL of sterilized deionized water. Control treatment: Each variety was subjected to one control treatment (CK), which did not include any waterlogging. The seeds were germinated as in the waterlogging treatment. The experimental and control treatments included five replicates, and each replicate consisted of 50 seeds. All the seeds were grown in a growth chamber with 25 °C, 75% relative humidity and a 16-h light/8-h dark cycle. The GR of the seeds (the number of germinated seeds) after 72 h of germination was calculated. Seeds were considered germinated if the radicle and the hypocotyl had emerged at least 2 mm from the seed coat. After 72 h of germination, we removed the radicle and coleoptile of the seeds; part of the samples were used for analysis of the seed anatomy, and the other samples were used for transcriptomic sequencing.

### Measurement of the GR and CH of wheat seeds

After 72 h of germination, the GR and the CH of the seeds in both the waterlogging and control treatments were measured.

### Structural anatomy of wheat seeds

To assess the anatomy of wheat seeds, the seeds were fixed in FAA (38% formaldehyde:glacial acetic acid:70% alcohol = 1:1:18 volume ratio) solution, sliced and embedded in paraffin. The slices were then processed as follows: Fist, the sections were sequentially placed in xylene I for 20 min, xylene II for 20 min, absolute ethanol I for 5 min, absolute ethanol II for 5 min, 75% alcohol for 5 min, and tap water for 5 min to deparaffinize the paraffin sections until dehydration. Second, the slices obtained using an ultramicrotome (Ultracut R, Leica, Germany) were sequentially stained with periodic acid, Schiff’s reagent, and naphthol yellow S (Wuhan Servicebio Technology Co., Ltd.). Third, the slices were dehydrated and mounted with anhydrous ethanol, xylene and neutral gum. Fourth, the slices were observed under a light microscope (DMLS, Leica, Solms, Germany) and photographed with a camera (TrueChrome II, Tucsen, China). Photoshop and Image-Pro Plus 6.0 (Media Cybernetics, Inc., Rockville, MD, USA) were used to count the numbers of amyloplasts based on the micrographs (three replicate seeds and 10 micrographs of each seed).

### Total RNA isolation, RNA library construction and transcriptome sequencing

To explore the response of wheat seeds to waterlogging stress, a transcriptome analysis of the seeds of three wheat varieties, ‘ZM22’, ‘BN207’ and ‘BN607’, under the waterlogging and control treatments was performed. A total of 18 samples (C72_ZM22, C72_BN207, C72_BN607, W72_ZM22, W72_BN207, and W72_BN607; each treatment was performed in triplicate) were first obtained by extracting total RNA from the samples using the TRIzol kit (Invitrogen, CA, USA). Total RNA was removed with RNase-free DNase I (Takara, Tokyo, Japan) to avoid genomic DNA contamination. To ensure that transcriptome sequencing was performed using high-quality samples, the purity, concentration, and integrity of RNA samples were determined using a NanoDrop, a Qubit 2.0, and an Agilent 2100, respectively. First, the mRNA was enriched with oligo (dT) magnetic beads, and fragmentation buffer was added to randomly disrupt the mRNA. The first cDNA strand was synthesized using mRNA as a template and a six-base random primer (random hexamers). To synthesize the second cDNA strand, the first cDNA strand was combined with buffer, dNTPs, RNase H, and DNA polymerase I. The purified double-stranded cDNA was subjected to end repair, A tailed and ligated to the sequencing adapter. Finally, fragment size selection was performed using AMPure XP beads, and a cDNA library was enriched by PCR. After the library was constructed, the concentration of the library and the insert size were detected using a Qubit 2.0 and an Agilent 2100, respectively. The average insert size of the paired-end libraries was 150 bp (±50 bp). After inspection of the library quality, transcriptome sequencing was performed using an Illumina NovaSeq™ 6000 (LC Sciences, San Diego, CA, USA) according to the standard protocols of the LC-Bio Technology Co., Ltd. (Hang Zhou, Zhejiang Province, China).

### Normalization and annotation of sequencing data

To ensure accurate and credible results, we preprocessed the raw data, which included the removal of sequencing adapters and low-quality sequencing data. The valid data were aligned to the reference genome of wheat (https://urgi.versailles.inra.fr/download/iwgsc/IWGSC_RefSeq_Assemblies/v1.0/), and statistical information was obtained based on gene location information specified by genome annotation files (GTF and GFF).

### Sequencing sequence statistics and quality control

The raw data generated by sequencing needs to be preprocessed. We used cutadapt to filter out unqualified sequences and obtain clean data [43]. The specific processing steps were as follows: (1) removing adapter-containing reads; (2) removing reads with a content ratio of N (N represents a base that cannot be determined) that is greater than 5% of the reads; (3) removing low-mass reads (mass value of Q < 10 bases accounting for more than 20% of the entire reads); and (4) analysing the amount of raw sequencing data, an effective amount of sequencing data, and the Q20, Q30, and GC contents.

### Analysis of DEGs and gene function annotation

The aligned reads from each sample were assembled using StringTie. The transcriptomes of all the samples were then reused to construct a comprehensive transcript utilising a Perl script. After the final generation of transcripts, StringTie and EdgeR were used to estimate the expression levels of all the transcripts. The expression levels of the genes were characterized by calculating FPKM values (fragments per kilobase of exon model per million mapped reads) [44], which eliminated the influences of the gene length and sequencing level on the calculation of gene expression. The DEGs in ‘ZM22’, ‘BN207’ and ‘BN607’ were identified by comparing the waterlogging and control treatments. The differentially expressed mRNAs with log2 (fold change) > 1 or log2 (fold change) < − 1 and with statistical significance (*p*-value < 0.05) were identified using the R package Ballgown [45]. The fold change represents the ratio of expression under the waterlogging and to that under the control treatment. An expression level dominance analysis was performed using EdgeR software [46], and a cluster analysis of the DEGs was performed using clustering software and Java Treeview. Gene functional annotations were based on the pear genome database and mapped to GO terms. GO enrichment analysis was performed using WEGO, and KEGG pathways were identified according to *p*-values and adjusted q values through a BLAST search against the KEGG database and subsequent mapping to KEGG pathways [47–52].

### Total RNA extraction, reverse transcription and qRT-PCR assays

Based on the target gene sequences, 10 gene-specific primer pairs were designed (Table S6). The RNA samples used for qRT-PCR analysis were aliquots of the samples used in the RNA-Seq experiments. The qRT-PCR assays were performed with the Primer Script RT Reagent Kit (Takara, Dalian, China) and the reference gene 18S (Gene ID: AJ272181.1) using cDNA from the transcriptome samples as the template. The total reaction volume for each qRT-PCR was 20 μL, which comprised 10 μL of SYBR Green PCR SuperMix (Vazyme Biotech Co., Ltd., Nanjing, China), 0.4 μL of each primer, 2 μL of cDNA, 0.4 μL of passive reference dye and 6.8 μL of double-distilled water. The PCR conditions were as follows: 95 °C for 10 s and 40 cycles of 95 °C for 5 s and 60 °C for 30 s. the qRT-PCRs were performed using an ABI Step One Plus. The qRT-PCR data were obtained from technical replicates with error bars and are presented as the means ± SEs (*n* = 3). One sample constitutes a mixture of three seeds. The relative expression was calculated using the 2^−ΔΔCt^ method [53].

### Statistical analysis

All statistical analyses were performed with SPSS 18.0 (SPSS Inc., Chicago, IL, USA) and Microsoft Excel (Microsoft Corporation, Redmond, WA, USA). The data were analysed by one-way analysis of variance (ANOVA). Mean separations were performed by Duncan’s multiple range tests. OriginPro 8.1 (Origin Inc., Chicago, IL, USA) was used to draw the figures. Differences with *p* < 0.05 were considered significant.

## Supplementary information

**Additional file 1: Table S1.** Summary of the RNA-Seq data collected from the seeds of three wheat varieties (ZM22, BN207 and BN607) under the waterlogging and control treatments.

**Additional file 2: Table S2.** Summary of the genome alignment distribution of the RNA-Seq data.

**Additional file 3: Table S3.** Summary of the GO results of the DEGs.

**Additional file 4: Table S4.** KEGG enrichment analysis of the DEGs.

**Additional file 5: Table S5.** Statistics of DEGs that exhibit different expression patterns.

**Additional file 6: Table S6.** List of primer sequences used for qPCR analysis.

**Additional file 7: Table S7.** Cluster analysis of the DEGs.

**Additional file 8: Figure S1.** Principal component analysis (PCA) f the transcription levels in the seeds of three wheat varieties (ZM22, BN207 and BN607) under the waterlogging and control treatments. ZM22: Zhoumai 22, BN207: Bainong 207, BN607: Bainong 607. W72_ZM22, W72_BN207 and W72_BN607 refer to ZM22, BN207 and BN607 under the waterlogging treatment, respectively. C72_ZM22, C72_BN207 and C72_BN607 refer to ZM22, BN207 and BN607 under the control treatment, respectively.

**Additional file 9: Figure S2.** GO enrichment of DEGs in the seeds of three wheat varieties (ZM22, BN207 and BN607) under the waterlogging and control treatments. W72_ZM22, W72_BN207 and W72_BN607 refer to ZM22, BN207 and BN607 under the waterlogging treatment, respectively. C72_ZM22, C72_BN207 and C72_BN607 refer to ZM22, BN207 and BN607 under the control treatment, respectively.

**Additional file 10: Figure S3.** KEGG enrichment factor analysis of DEGs in the seeds of three wheat varieties (ZM22, BN207 and BN607) under the waterlogging and control treatments. W72_ZM22, W72_BN207 and W72_BN607 refer to ZM22, BN207 and BN607 under the waterlogging treatment, respectively. C72_ZM22, C72_BN207 and C72_BN607 refer to ZM22, BN207 and BN607 under the control treatment, respectively.

**Additional file 11: Figure S4.** RT-qPCR validations of the RNA-seq data. Expression profiling of 10 candidate genes of the seeds of three wheat varieties (ZM22, BN207 and BN607) under the waterlogging and control treatments. W72_ZM22, W72_BN207 and W72_BN607 refer to ZM22, BN207 and BN607 under the waterlogging treatment, respectively. C72_ZM22, C72_BN207 and C72_BN607 refer to ZM22, BN207 and BN607 under the control treatment, respectively.

## Data Availability

The datasets supporting the results described in this article are included within the article and its additional file. All raw sequence reads have been deposited in NCBI’s Gene Expression Omnibus and are accessible under the GEO Series accession number GSE144554 (https://www.ncbi.nlm.nih.gov/geo/query/acc.cgi?acc=GSE144554).

## Abbreviations

ZM22: Zhoumai 22
BN207: Bainong 207
BN607: Bainong 607
DEGs: Differentially Expressed Genes
ADH: Alcohol Dehydrogenase
GO: Gene Ontology
mRNA: Messenger RNA
KEGG: Kyoto Encyclopedia of Genes and Genomes
PCA: Principal Component Analysis
qRT-PCR: Quantitative Real-time PCR
FPKM: Fragments Per Kilobase Per Million Mapped Reads
NCBI: National Center for Biotechnology Information
PDC: Pyruvate Decarboxylase
LDH: Lactate Dehydrogenase
GR: Germination Rate
CH: Coleoptile Height
FAA: 38% Formaldehyde-Acetic acid-70% Alcohol
