# Supplementary figures and images for: Transcriptomic and anatomic profiling reveal the germination process of different wheat varieties in response to waterlogging stress
Source: BMC Genet. 2020 Aug 28;21:93. doi: 10.1186/s12863-020-00901-y (PMC7456028; doi:10.1186/s12863-020-00901-y)

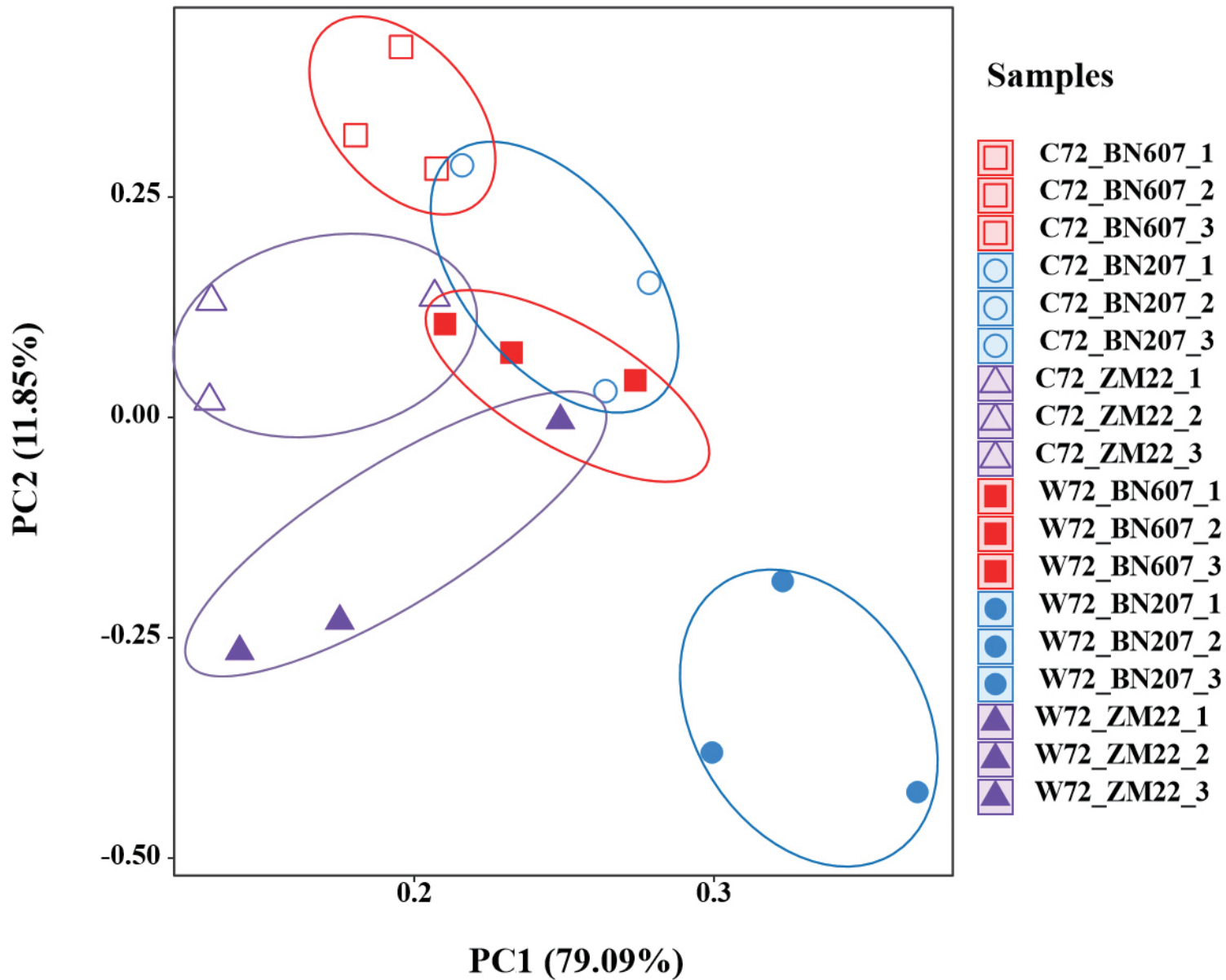

Supplement: Supplementary file 8 — Additional file 8: Figure S1. Principal component analysis (PCA) f the transcription levels in the seeds of three wheat varieties (ZM22, BN207 and BN607) under the waterlogging and control treatments. ZM22: Zhoumai 22, BN207: Bainong 207, BN607: Bainong 607. W72_ZM22, W72_BN207 and W72_BN607 refer to ZM22, BN207 and BN607 under the waterlogging treatment, respectively. C72_ZM22, C72_BN207 and C72_BN607 refer to ZM22, BN207 and BN607 under the control treatment, respectively. [file 12863_2020_901_MOESM8_ESM.pdf]

A

## W72\_ZM22 vs C72\_ZM22

pathway\_name

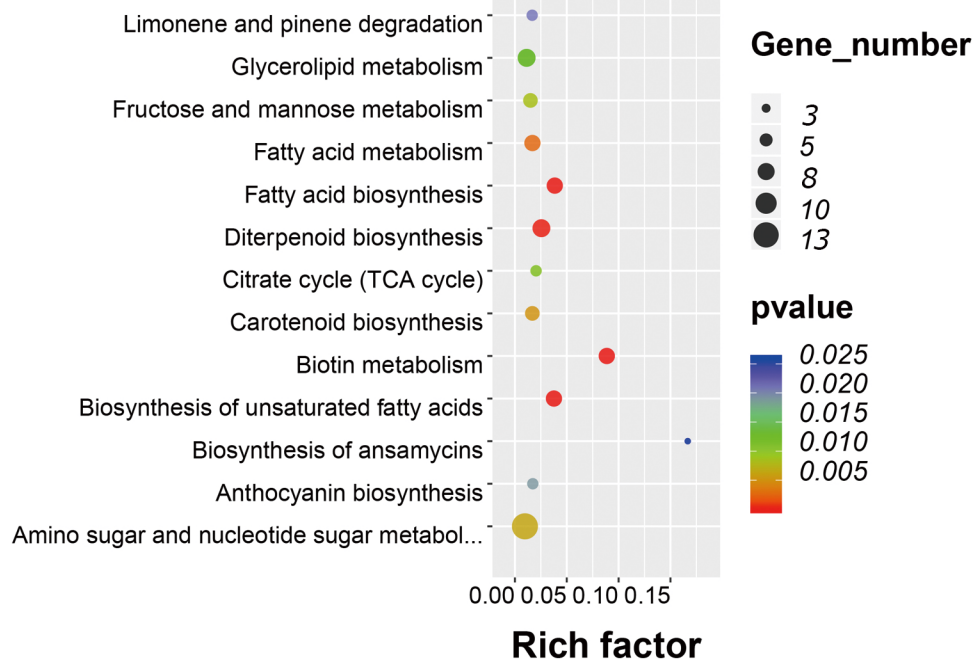

B

## W72\_BN207 vs C72\_BN207

pathway\_name

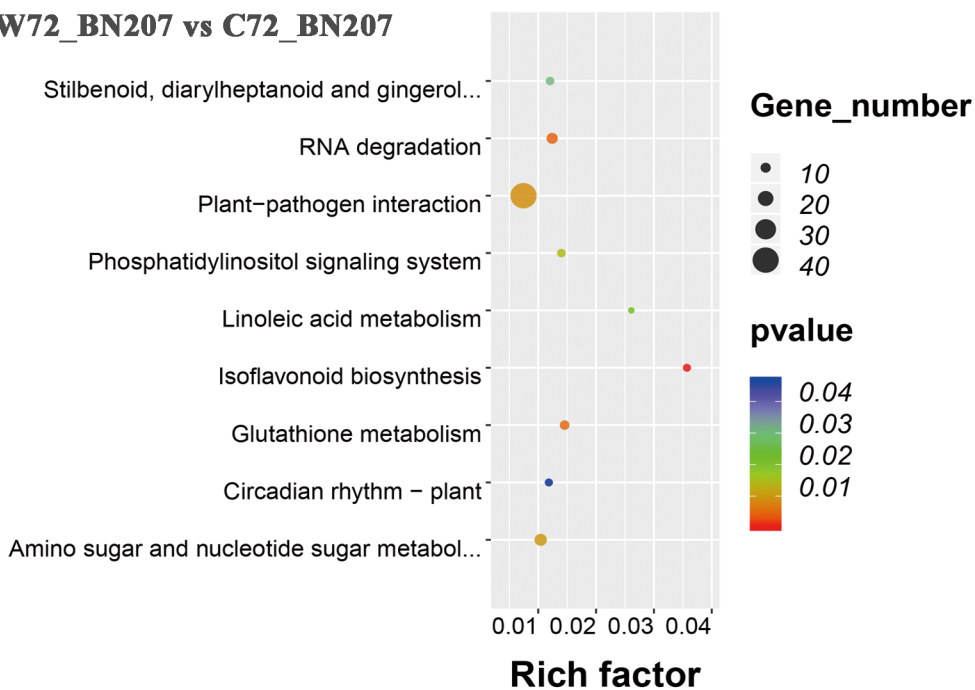

C

## W72\_BN607 vs C72\_BN607

pathway\_name

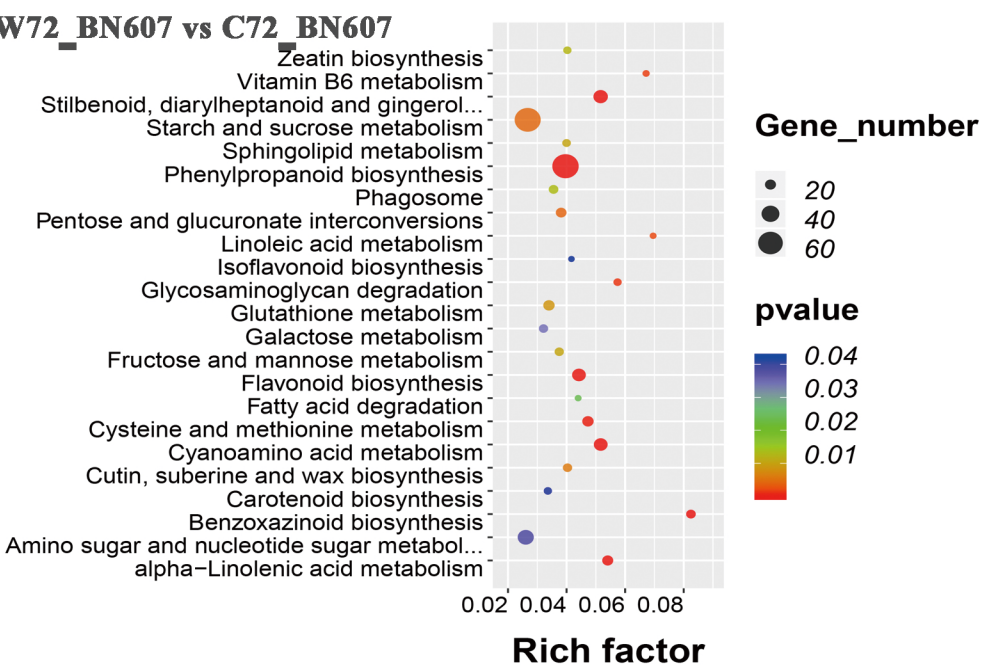

Supplement: Supplementary file 10 — Additional file 10: Figure S3. KEGG enrichment factor analysis of DEGs in the seeds of three wheat varieties (ZM22, BN207 and BN607) under the waterlogging and control treatments. W72_ZM22, W72_BN207 and W72_BN607 refer to ZM22, BN207 and BN607 under the waterlogging treatment, respectively. C72_ZM22, C72_BN207 and C72_BN607 refer to ZM22, BN207 and BN607 under the control treatment, respectively. [file 12863_2020_901_MOESM10_ESM.pdf]

*ADH2*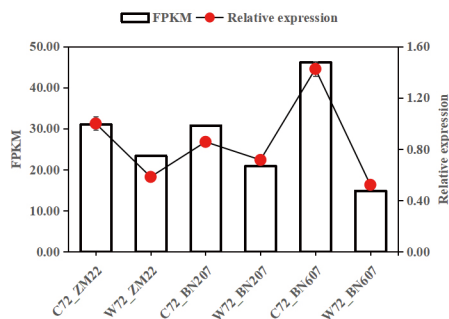*GT7*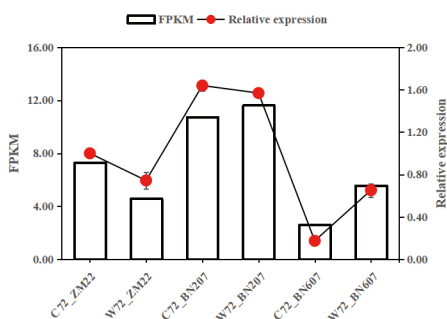*SSII-3*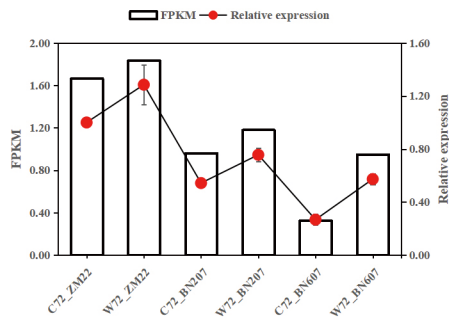*GAUT4*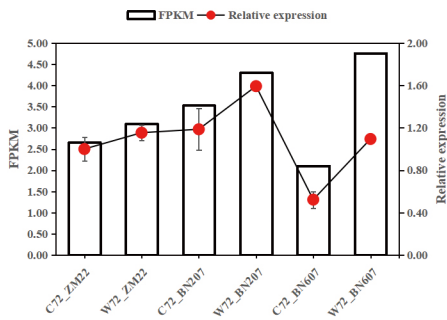*CRK10*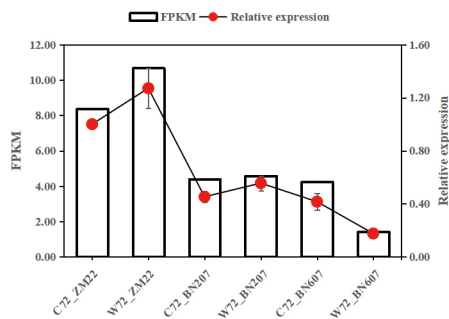*Cht4*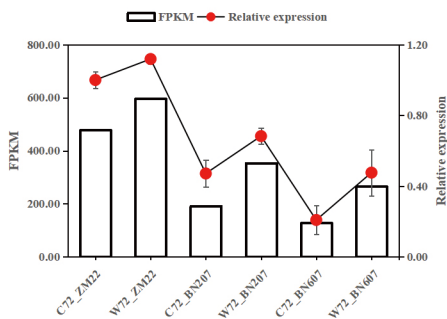*ASD1*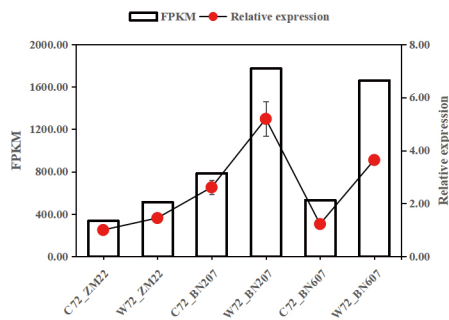*GUX1*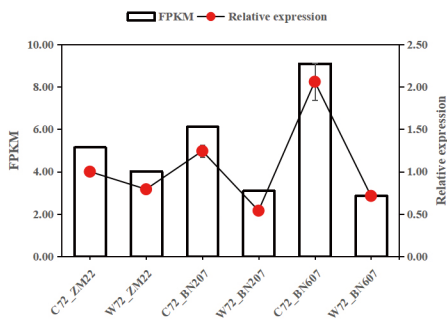*DGD11*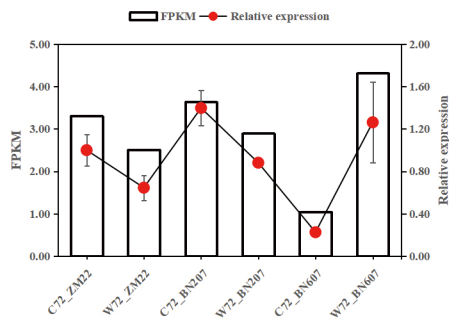*PAH1*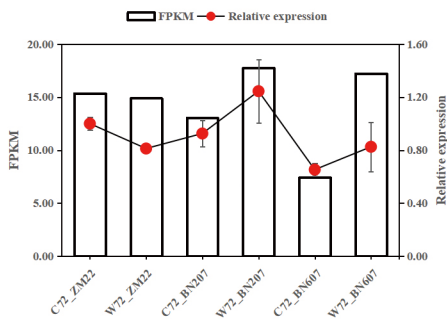

Supplement: Supplementary file 11 — Additional file 11: Figure S4. RT-qPCR validations of the RNA-seq data. Expression profiling of 10 candidate genes of the seeds of three wheat varieties (ZM22, BN207 and BN607) under the waterlogging and control treatments. W72_ZM22, W72_BN207 and W72_BN607 refer to ZM22, BN207 and BN607 under the waterlogging treatment, respectively. C72_ZM22, C72_BN207 and C72_BN607 refer to ZM22, BN207 and BN607 under the control treatment, respectively. [file 12863_2020_901_MOESM11_ESM.pdf]
