# Supplementary material for: Transcriptomic and anatomic profiling reveal the germination process of different wheat varieties in response to waterlogging stress
Source: BMC Genet. 2020 Aug 28;21:93. doi: 10.1186/s12863-020-00901-y (PMC7456028; doi:10.1186/s12863-020-00901-y)
Supplement: Supplementary file 9 — Additional file 9: Figure S2. GO enrichment of DEGs in the seeds of three wheat varieties (ZM22, BN207 and BN607) under the waterlogging and control treatments. W72_ZM22, W72_BN207 and W72_BN607 refer to ZM22, BN207 and BN607 under the waterlogging treatment, respectively. C72_ZM22, C72_BN207 and C72_BN607 refer to ZM22, BN207 and BN607 under the control treatment, respectively. [file 12863_2020_901_MOESM9_ESM.pdf]

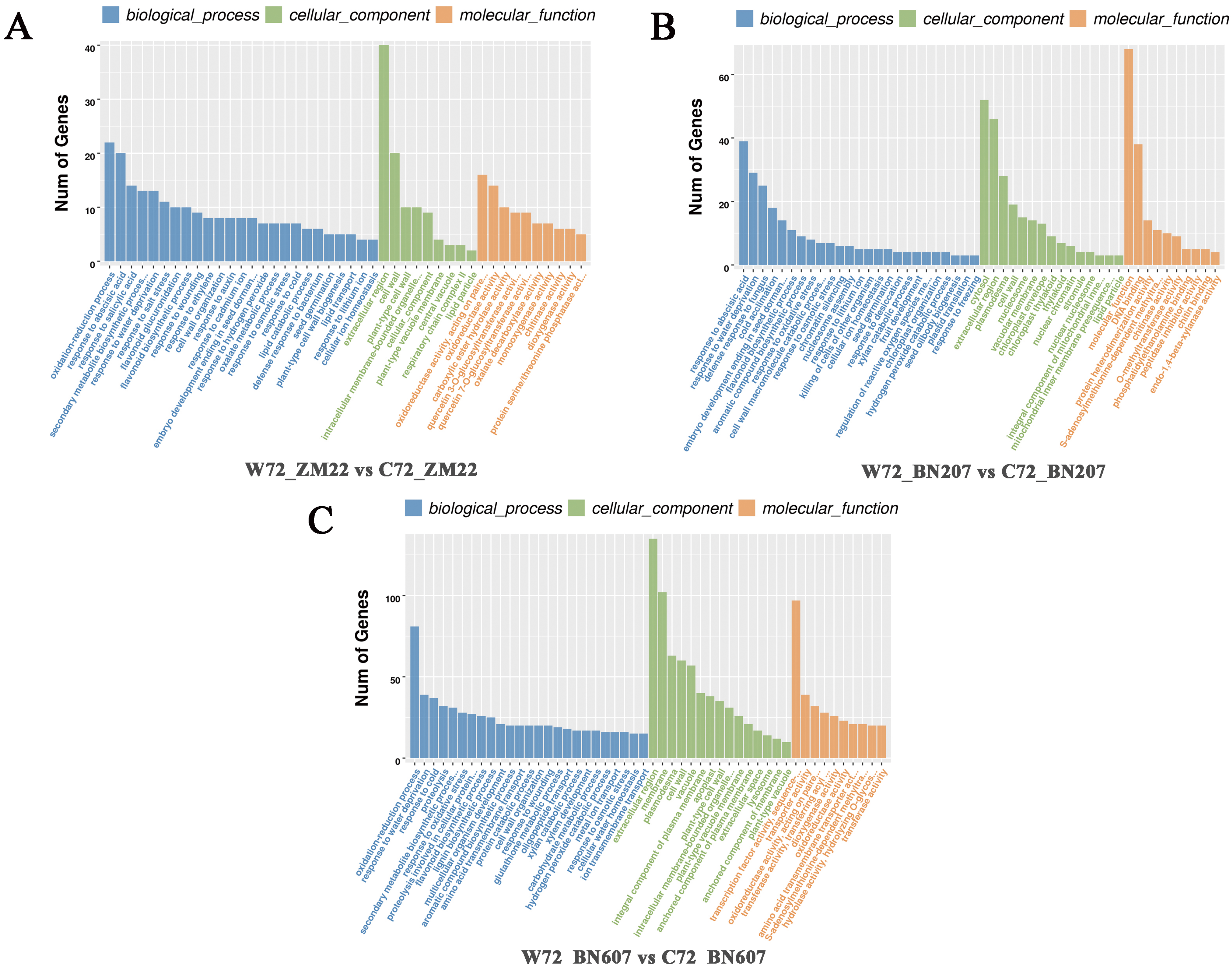
A
biological_process
cellular_component
molecular_function
Num of Genes
0
10
20
30
40
oxidation-reduction process
response to abscisic acid
response to salicylic acid
secondary metabolite biosynthetic proces...
response to water deprivation
response to salt stress
flavonoid glucuronidation
flavonoid biosynthetic process
response to wounding
response to ethylene
cell wall organization
response to auxin
response to cadmium ion
embryo development ending in seed dorman...
response to hydrogen peroxide
oxalate metabolic process
response to osmotic stress
response to cold
lipid catabolic process
defense response to bacterium
seed germination
plant-type cell wall biogenesis
lipid transport
response to lithium ion
cellular ion homeostasis
extracellular region
cell wall
plant-type cell wall
intracellular membrane-bounded organelle...
cellular_component
plant-type vacuole membrane
central vacuole
respiratory chain complex II
lipid particle
oxidoreductase activity, acting on paire...
oxidoreductase activity
carboxylic ester hydrolase activity
quercetin 3-O-glucosyltransferase activi...
quercetin 7-O-glucosyltransferase activi...
oxalate decarboxylase activity
monooxygenase activity
chitinase activity
dioxygenase activity
protein serine/threonine phosphatase act...
W72_ZM22 vs C72_ZM22
B
biological_process
cellular_component
molecular_function
Num of Genes
0
20
40
60
response to abscisic acid
response to water deprivation
defense response to fungus
cold acclimation
embryo development ending in seed dorman...
flavonoid biosynthetic process
aromatic compound biosynthetic process
response to oxidative stress
cell wall macromolecule catabolic proces...
response to osmotic stress
chromatin silencing
nucleosome assembly
response to lithium ion
killing of cells of other organism
cellular ion homeostasis
seed germination
response to desiccation
xylan catabolic process
fruit development
regulation of reactive oxygen species me...
chloroplast organization
hydrogen peroxide catabolic process
seed oilbody biogenesis
plastid translation
response to freezing
cytosol
extracellular region
plasmodesma
cell wall
nucleosome
vacuolar membrane
chloroplast envelope
chloroplast thylakoid
thylakoid
nuclear chromatin
stromule
nuclear nucleosome
integral component of mitochondrial inne...
mitochondrial inner membrane presequence...
lipid particle
molecular_function
DNA binding
protein heterodimerization activity
S-adenosylmethionine-dependent methyltra...
chitinase activity
O-methyltransferase activity
phosphatidylethanolamine binding
peptidase inhibitor activity
chitin binding
endo-1,4-beta-xylanase activity
W72_BN207 vs C72_BN207
C
biological_process
cellular_component
molecular_function
Num of Genes
0
50
100
oxidation-reduction process
response to water deprivation
response to cold
proteolysis
secondary metabolite biosynthetic proces...
response to oxidative stress
proteolysis involved in cellular protein...
flavonoid biosynthetic process
lignin biosynthetic process
multicellular organism development
aromatic compound biosynthetic process
amino acid transmembrane transport
protein catabolic process
cell wall organization
response to wounding
glutathione metabolic process
oligopeptide transport
xylan catabolic process
xylem development
carbohydrate metabolic process
hydrogen peroxide catabolic process
metal ion transport
response to osmotic stress
cellular water homeostasis
ion transmembrane transport
extracellular region
membrane
plasmodesma
cell wall
vacuole
integral component of plasma membrane
apoplast
intracellular membrane-bounded organelle...
plant-type cell wall
plant-type vacuole membrane
anchored component of plasma membrane
extracellular space
lysosome
anchored component of membrane
plant-type vacuole
transcription factor activity, sequence...
transporter activity
oxidoreductase activity, acting on paire...
transferase activity, transferring acyl...
dioxygenase activity
oxidoreductase activity
amino acid transmembrane transporter act...
S-adenosylmethionine-dependent methyltra...
hydrolase activity, hydrolyzing O-glycos...
transferase activity
W72_BN607 vs C72_BN607
